# Epigenome-wide association study of Alzheimer’s disease replicates 22 differentially methylated positions and 30 differentially methylated regions

**DOI:** 10.1186/s13148-020-00944-z

**Published:** 2020-10-17

**Authors:** Qingqin S. Li, Yu Sun, Tania Wang

**Affiliations:** 1 Neuroscience, Janssen Research & Development, LLC, 1125 Trenton-Harbourton Road, Titusville, NJ 08560 USA; 2 AccuraScience, LLC, Johnston, IA USA; 3 Present Address: Discovery Science, Janssen Research & Development, LLC, Spring House, PA USA; 4 Present Address: Center for Medical Genetics and Hunan Key Laboratory of Medical Genetics, School of Life Sciences, Central South University, Changsha, 410083 China; 5 Present Address: Beijing Institutes of Life Science, Chinese Academy of Sciences, Beijing, 100101 China

**Keywords:** Epigenetics, EWAS, DMP, DMR

## Abstract

**Background:**

Growing evidence shows that epigenetic modifications play a role in Alzheimer’s disease (AD). We performed an epigenome-wide association study (EWAS) to evaluate the DNA methylation differences using postmortem superior temporal gyrus (STG) and inferior frontal gyrus (IFG) samples.

**Results:**

Samples from 72 AD patients and 62 age-matched cognitively normal controls were assayed using Illumina^©^ Infinium MethylationEPIC BeadChip. Five and 14 differentially methylated positions (DMPs) associated with pathology (i.e., Braak stage) with p value less than Bonferroni correction threshold of 6.79 × 10^–8^ in the STG and IFG were identified, respectively. These cytosine–phosphate–guanine (CpG) sites included promoter associated cg26263477 annotated to *ABCA7* in the STG (*p* = 1.21 × 10^–11^), and cg14058329 annotated to the *HOXA5/HOXA3/HOXA-AS3* gene cluster (*p* = 1.62 × 10^–9^) and cg09448088 (*p* = 3.95 × 10^–9^) annotated to *MCF2L* in the IFG. These genes were previously reported to harbor DMPs and/or differentially methylated regions (DMRs). Previously reported DMPs annotated to *RMGA*, *GNG7*, *HOXA3*, *GPR56*, *SPG7*, *PCNT*, *RP11-961A15.1, MCF2L*, *RHBDF2*, *ANK1*, *PCNT*, *TPRG1*, and *RASGEF1C* were replicated (*p* < 0.0001). One hundred twenty-one and 173 DMRs associated with pathology in the STG and IFG, respectively, were additionally identified. Of these, DMRs annotated to 30 unique genes were also identified as significant DMRs in the same brain region in a recent meta-analysis, while additional DMRs annotated to 12 genes were reported as DMRs in a different brain region or in a cross-cortex meta-analysis. The significant DMRs were enriched in promoters, CpG islands, and exons in the genome. Gene set enrichment analysis of DMPs and DMRs showed that gene sets involved in neuroinflammation (e.g., microglia differentiation), neurogenesis, and cognition were enriched (false discovery rate (FDR) < 0.05).

**Conclusions:**

Twenty-two DMPs and 30 DMRs associated with pathology were replicated, and novel DMPs and DMRs were discovered.

## Introduction

Dementia refers to conditions of memory loss and other cognitive decline serious enough to interfere with daily life. Alzheimer's disease (AD) is the most common cause of dementia and accounts for 50–75% of dementia cases [1]. While genetic studies have identified familial risk factors such as *APP*, *PSEN1*, and *PSEN2* that are involved with amyloid-β production, they only account for a small fraction of patients with early onset AD [2]. Most patients with AD acquire the disease late in life (i.e., age of onset > 65 years). Genome-wide association studies (GWAS) have identified dozens of loci for late onset AD (LOAD) [3–9]. Most of the variants confer risk with relatively small effect size, except for *APOE* variants with modest effect size. Next generation sequencing (NGS) enables rare variant analysis to further identify genes such as *TREM2* playing a critical role in AD [9–11]. Growing evidence shows that epigenetic modifications also play a role in AD onset and progression [12–15]. Epigenetic modification could be detected by bisulfite conversion, a method that assesses the degree of DNA methylation present as it converts unmethylated cytosine to uracil (and to thymine through PCR), while 5-methylcytosine (5-mC) and 5-hydroxymethylcytosine (5-hmC) are not converted (and thus remain as cytosine in PCR). As such, bisulfite treatment of DNA allows the differentiation between cytosine and the modified versions of cytosine (5-mC/5-hmC) through downstream assay techniques. Oxidative bisulfite conversion may further distinguish 5-mC from 5-hmC [16]. Epigenome-wide association studies (EWAS) using bisulfite conversion approaches coupled with the Illumina Infinium® HumanMethylation450 BeadChip have demonstrated robust and reproducible differences in total DNA methylation at a number of loci in AD brain [17–22], including ankyrin 1 (*ANK1*), *ABCA7, BIN1, TREM2,* and the *HOXA* and *HOXB* gene clusters. Notably, a recent meta-analysis using samples from 6 cohorts identified a total of 220 DMPs in a cross-cortex meta-analysis [21].

Volumetric measurements of specific regions of the cortex from AD patients reveal anatomical regions with severe, moderate, or mild/no atrophy. Severe atrophy occurs in medial temporal lobe structures as well as in inferior temporal and superior and middle frontal cortices, while moderate atrophy takes place in the superior temporal gyrus (STG) and no atrophy is noted in the inferior frontal lobes [23]. While there is no significant increase in total (intra- and extracellular) neurofibrillary tangles (NFTs), moderate neuronal loss and evidence of oxidative stress are observed in the STG [24–28]. The STG could therefore be a surrogate for an earlier stage compared to the most severely affected regions, while inferior frontal gyrus (IFG) could represent the earliest stage in the disease course. In this EWAS study, total methylation patterns from the STG and IFG were interrogated using Infinium® MethylationEPIC BeadChip containing ~ 850 K CpG probes, doubling the density of the Infinium® HumanMethylation450 BeadChip used in earlier studies [17, 18, 20, 29]. DMPs and DMRs associated with pathology were identified. Additionally, we performed a replication study using this study data set to replicate the findings from a recent EWAS meta-analysis [21]. Gene set enrichment and over representation analyses were performed to provide insight into coherent biological pathways and processes.

## Results

### Postmortem brain tissue epigenetic profiling and DMP analysis results

Demographic and clinical characteristics are provided in Table 1. The mean Pearson correlations of methylation levels for all possible subject pairs were 0.986 and 0.984 for the STG and IFG samples, respectively, indicating that the majority of the CpG sites did not show significant differences in DNA methylation levels. The estimated proportion of NeuN^+^ cells (primarily neurons) showed no significant differences between AD patients and cognitively normal controls (Wilcoxon rank-sum test *p* value = 0.504 and 0.159 in the STG and IFG, respectively).

**Table 1:** Demographic and clinical characteristics of the samples used in the EPIC array assay

Brain region	STG			IFG		
Clinical diagnosis	Cognitively normal	Alzheimer’s disease	p**	Cognitively normal	Alzheimer’s Disease	p**
Sample size	*N* = 60	*N* = 67		*N* = 57	*N* = 60	
Age at death (year), mean (SD)	80.65 (6.94)	81.00 (7.09)	0.717	81.00 (6.63)	81.13 (6.40)	0.967
Sex, male *n* (%)	37 (61.7)	39 (58.2)	0.692	36 (63.2)	35 (58.3)	0.593
PMI (hour), Mean (SD)	3.24 (2.02)	3.06 (1.60)	0.837	3.25 (2.05)	3.15 (1.69)	0.846
Estimated NeuN^+^ (%) Mean (SD)	30.3 (13.1)	29.6 (11.3)	0.504	24.5 (12.3)	28.1 (9.9)	0.159
Estimated NeuN^−^ (%), Mean (SD)	68.6 (14.1)	69.3 (12.7)	0.570	69.0 (15.4)	64.1 (12.0)	0.095
NIA-Reagan criteria [72], *n*						
Criteria not met	59			56		
Not AD	1			1		
Low		2			2	
Intermediate		16			17	
High		49			41	
Semiquantitative measure of neuritic plaques CERAD score [73], *n*						
Criteria not met	6			6		
Not AD	33			31		
Possible AD	21			20		
Probable AD		8			8	
Definite AD		59			52	
Braak stage, *n*						
I	15			12		
II	14	2		14	2	
III	20	4		21	4	
IV	11	12		10	13	
V		28			21	
VI		21			20	
APOE genotype, n*						
ε2/ε2	1	0		2	0	
ε2/ε3	10	3		10	1	
ε3/ε3	32	26		30	26	
ε3/ε4	16	33		15	27	
ε2/ε4	0	1		0	1	
ε4/ε4	1	3		0	4	

SD: standard deviation; STG: superior temporal gyrus (BA22); IFG: inferior frontal gyrus (BA44); PMI: postmortem interval; CERAD [73]: Consortium to Establish a Registry for Alzheimer’s Disease

*1 STG and 1 IFG sample, respectively, has missing *APOE* genotype

**Wilcoxon rank-sum test for continuous variables; Chi-squared test for categorical variables

Epigenome-wide association studies are known to be prone to significant inflation and bias of test statistics. Lambda (*λ*) inflation factors were 1.54 and 1.11 for the initial EWAS in the STG and IFG, respectively, suggesting the presence of inflation in test statistics (Additional file 1: Figure S1 for QQ plots). A Bayesian method based on estimation of the empirical null distribution as implemented in BACON [30] was used to control bias and inflation in EWAS. After BACON correction, *λ* values for both EWAS were less than 1.05 in this study. All results reported in this study are after BACON correction. Manhattan plots are also available in Additional file 1: Figure S2.

**Fig. 1:**
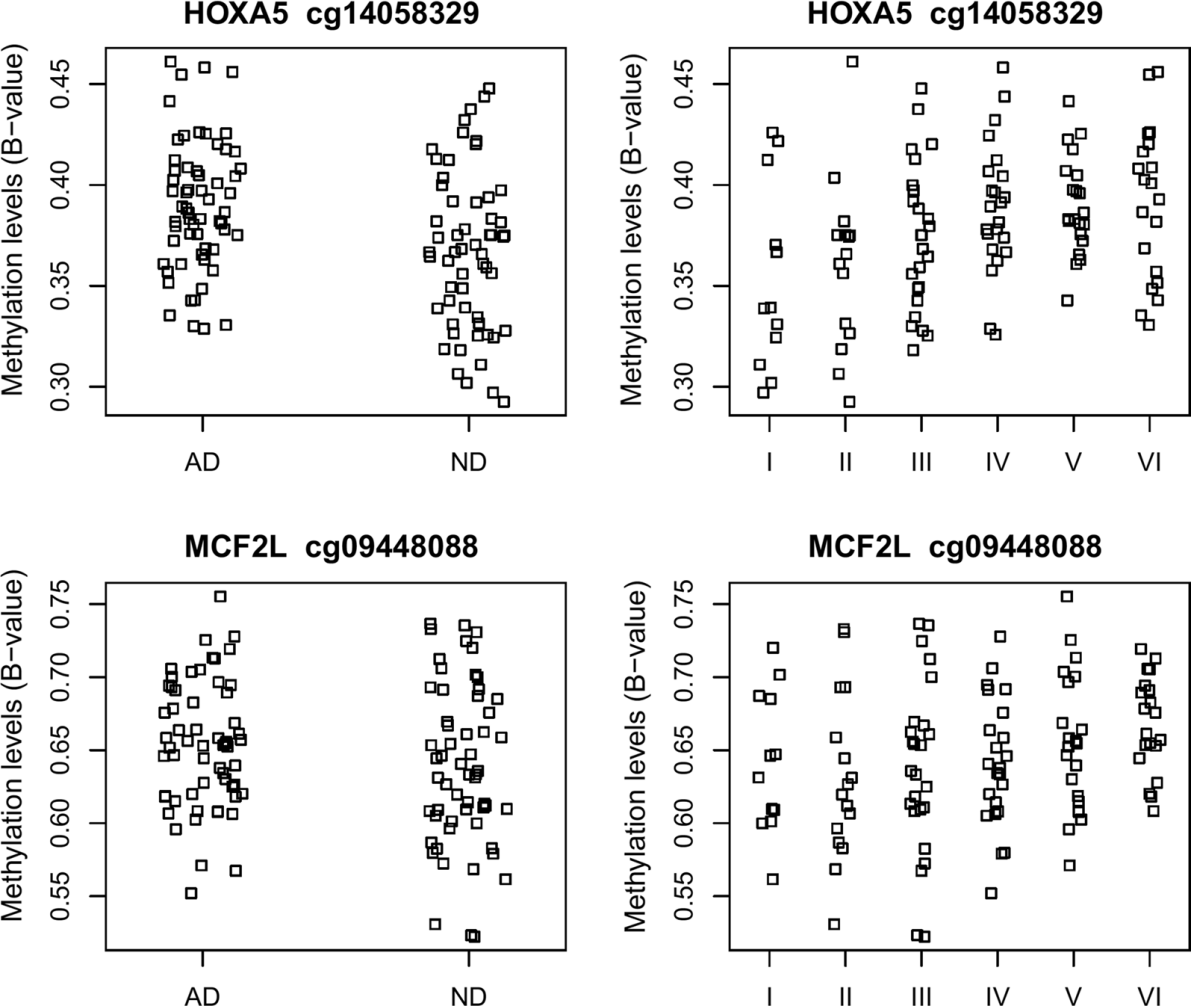
DMP associations with diagnosis and pathology. The methylation level as measured by B value for probe cg14058329 annotated to *HOXA5* was plotted again AD diagnosis (**a**) and Braak stage (**b**). Similarly, B value for probe cg09448088 annotated to *MCF2L* was plotted again diagnosis (**c**) and Braak stage (**d**). In both cases, hypermethylation was observed in later Braak stage than earlier stage

Five CpGs were associated with pathology in the STG passing Bonferroni correction threshold of 6.79 × 10^–8^, including cg26263477 annotated to *ABCA7* (*p* = 1.21 × 10^–11^, Table 2), a gene known to harbor an *AD* susceptibility genetic variant and DMP [5, 31]. In addition, fourteen CpG probes were associated with pathology in the IFG, including CpG probes cg14058329 annotated to the *HOXA5/HOXA3/HOX-AS3* gene cluster (*p* = 1.62 × 10^–9^) and cg09448088 (*p* = 3.95 × 10^–9^) annotated to *MCF2L* (Fig. 1). cg09448088 was recently reported as a significant DMP in an cross-cortex Braak stage EWAS meta-analysis [21], and the *HOXA* gene cluster was reported to harbor DMPs and DMRs associated with Braak stage [20, 21]. There is no overlap between the study-wide significant findings between these two brain regions.

**Table 2:** Replicated DMPs associated with Braak stage

Name	Chr	Pos	Gene annotation	Strand	Relation to Island	UCSC RefGene Group	This study				Smith et al. meta-analysis		
							STG		IFG		ES	*p* value	Brain region
							beta	*p* value	beta	*p* value			
cg15645660	1	55,247,356		+	Island	Body	0.09	1.03E−03	0.09	2.28E−05	0.04	2.12E−08	Prefrontal Cortex
cg15645660	1	55,247,356		+	Island	Body	0.09	1.03E−03	0.09	2.28E−05	0.03	4.67E−10	Cross Cortex
cg17104258	1	1.67E+08		−	Island	Body	− 0.12	8.72E−03	− 0.22	5.99E−06	− 0.02	6.27E−11	Cross Cortex
cg17104258	1	1.67E+08		−	Island	Body	− 0.12	8.72E−03	− 0.22	5.99E−06	− 0.03	2.60E−08	TG
cg12307200	3	1.89E+08	*TPRG1;TPRG1-AS1*	+	N Shore		− 0.05	7.18E−04	− 0.04	8.65E−05	− 0.02	2.19E−11	Prefrontal Cortex
cg12307200	3	1.89E+08	*TPRG1;TPRG1-AS1*	+	N Shore		− 0.05	7.18E−04	− 0.04	8.65E−05	− 0.02	2.72E−12	TG
cg12307200	3	1.89E+08	*TPRG1;TPRG1-AS1*	+	N Shore		− 0.05	7.18E−04	− 0.04	8.65E−05	− 0.02	4.48E−16	Cross Cortex
cg11964461	5	1.8E+08	*RASGEF1C*	−	OpenSea	5′UTR	0.01	1.58E−01	0.03	1.01E−04	0.01	9.48E−08	Cross Cortex
cg00921266	7	27,153,663	*HOXA3;HOXA-AS2;HOXA3*	−	N Shore	5′UTR;TSS200	0.11	8.39E−05	0.04	2.01E−01	0.03	8.94E−10	Prefrontal Cortex
cg00921266	7	27,153,663	*HOXA3;HOXA-AS2;HOXA3*	−	N Shore	5′UTR;TSS200	0.11	8.39E−05	0.04	2.01E−01	0.02	9.25E−09	Cross Cortex
cg05066959	8	41,519,308	*ANK1*	−	OpenSea	TSS1500;Body	0.10	2.91E−02	0.10	2.04E−05	0.02	1.45E−12	Cross Cortex
cg05066959	8	41,519,308	*ANK1*	−	OpenSea	TSS1500;Body	0.10	2.91E−02	0.10	2.04E−05	0.04	4.58E−13	TG
cg05066959	8	41,519,308	*ANK1*	−	OpenSea	TSS1500;Body	0.10	2.91E−02	0.10	2.04E−05	0.03	7.35E−10	Prefrontal Cortex
cg05066959	8	41,519,308	*ANK1*	−	OpenSea	TSS1500;Body	0.10	2.91E−02	0.10	2.04E−05	0.06	2.93E−09	EC
cg23968456	10	73,521,631		−	OpenSea	Body	0.11	1.95E−02	0.09	2.37E−05	0.01	1.09E−09	Cross Cortex
cg23968456	10	73,521,631		−	OpenSea	Body	0.11	1.95E−02	0.09	2.37E−05	0.01	1.06E−09	TG
cg10469774	11	44,642,932		−	OpenSea		0.05	2.23E−03	0.07	4.77E−05	0.01	7.26E−09	Cross Cortex
cg07883124	13	1.14E+08	*MCF2L*	−	Island	Body;1stExon	0.03	3.52E−02	0.05	8.57E−06	0.02	9.10E−13	Cross Cortex
cg07883124	13	1.14E+08	*MCF2L*	−	Island	Body;1stExon	0.03	3.52E−02	0.05	8.57E−06	0.02	6.40E−08	Prefrontal Cortex
cg09448088	13	1.14E+08		−	Island	Body	0.02	8.69E−02	0.06	3.95E−09	0.01	1.04E−08	Cross Cortex
cg26127778	15	93,617,141	*RGMA*	−	Island	1stExon;TSS1500;Body;5′UTR	− 0.05	3.52E−05	− 0.03	1.88E−03	− 0.02	4.88E−08	TG
cg17400113	15	93,617,146	*RGMA*	−	Island	1stExon;TSS1500;Body;5′UTR	− 0.03	1.23E−03	− 0.03	1.75E−05	− 0.01	1.15E−08	Cross Cortex
cg17400113	15	93,617,146	*RGMA*	−	Island	1stExon;TSS1500;Body;5′UTR	− 0.03	1.23E−03	− 0.03	1.75E−05	− 0.02	3.75E−08	TG
cg09109520	16	57,673,258	*GPR56*	−	OpenSea	5′UTR;1stExon	0.06	9.14E−05	0.03	1.39E−01	0.02	9.00E−11	Cross Cortex
cg09109520	16	57,673,258	*GPR56*	−	OpenSea	5′UTR;1stExon	0.06	9.14E−05	0.03	1.39E−01	0.02	2.53E−10	Prefrontal Cortex
cg03169557	16	89,598,950	*SPG7*	+	OpenSea	Body	0.13	4.99E−02	0.21	1.33E−06	0.01	3.15E−08	Prefrontal Cortex
cg03169557	16	89,598,950	*SPG7*	+	OpenSea	Body	0.13	4.99E−02	0.21	1.33E−06	0.02	2.71E−10	TG
cg03169557	16	89,598,950	*SPG7*	+	OpenSea	Body	0.13	4.99E−02	0.21	1.33E−06	0.01	5.41E−11	Cross Cortex
cg03169557	16	89,598,950	*SPG7*	+	OpenSea	Body	0.13	4.99E−02	0.21	1.33E−06	0.02	4.41E−09	EC
cg19803550	17	1,637,391	*RP11-961A15.1*	+	Island	Body	0.14	1.91E−02	0.13	1.67E−05	0.01	1.06E−08	Cross Cortex
cg19803550	17	1,637,391	*RP11-961A15.1*	+	Island	Body	0.14	1.91E−02	0.13	1.67E−05	0.01	3.68E−08	Prefrontal Cortex
cg05810363	17	74,475,270		−	Island	Body	0.12	6.05E−02	0.16	6.73E−07	0.03	2.25E−11	TG
cg05810363	17	74,475,270		−	Island	Body	0.12	6.05E−02	0.16	6.73E−07	0.02	2.01E−10	Cross Cortex
cg13076843	17	74,475,294		+	Island	Body	0.07	3.13E−02	0.10	1.02E−04	0.03	7.31E−10	Prefrontal Cortex
cg13076843	17	74,475,294		+	Island	Body	0.07	3.13E−02	0.10	1.02E−04	0.02	7.57E−13	Cross Cortex
cg13076843	17	74,475,294		+	Island	Body	0.07	3.13E−02	0.10	1.02E−04	0.03	2.97E−11	TG
cg12163800	17	74,475,355	*RHBDF2*	+	Island	Body	0.08	1.92E−03	0.10	9.19E−06	0.01	5.13E−09	Cross Cortex
cg12163800	17	74,475,355	*RHBDF2*	+	Island	Body	0.08	1.92E−03	0.10	9.19E−06	0.02	2.06E−08	Prefrontal Cortex
cg12163800	17	74,475,355	*RHBDF2*	+	Island	Body	0.08	1.92E−03	0.10	9.19E−06	0.02	5.85E−09	TG
cg12309456	17	74,475,402		−	Island	Body	0.09	2.46E−02	0.10	7.68E−06	0.02	1.33E−08	TG
cg12309456	17	74,475,402		−	Island	Body	0.09	2.46E−02	0.10	7.68E−06	0.01	4.09E−08	Cross Cortex
cg08481112	19	2,544,100	*GNG7*	+	S Shore	5′UTR	0.07	4.81E−05	0.03	7.27E−02	0.02	2.84E−08	Prefrontal Cortex
cg23449541	21	47,855,893	*PCNT*	−	Island	Body	0.04	3.16E−02	0.07	8.45E−06	0.01	7.49E−08	Cross Cortex
cg23449541	21	47,855,893	*PCNT*	−	Island	Body	0.04	3.16E−02	0.07	8.45E−06	0.02	9.43E−11	TG
cg00621289	21	47,855,916	*PCNT*	−	Island	Body	0.04	1.43E−02	0.06	2.51E−05	0.01	4.69E−11	Cross Cortex

Chr: chromosome; Pos: base pair position in reference to human genome hg19; Gene Annotation: gene annotation in reference to Gencode Basic V12; beta coefficient and *p* value was reported from the limma robust regression model for this study for each DMP, while the effect size (ES) and corresponding unadjusted *p* value from the inverse variance fixed effects meta-analysis model was shown for the Smith et al. meta-analysis. In addition, ES from the Smith et al. meta-analysis has been multiplied by six to demonstrate the difference between Braak stage 0 and Braak stage VI samples. This was not done for this study

**Fig. 2:**
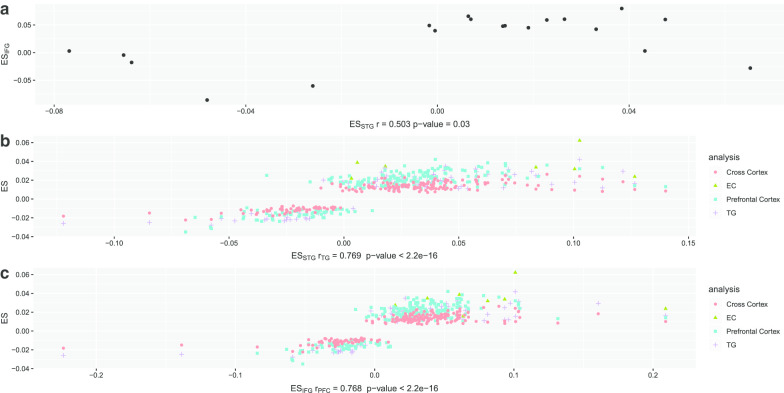
Correlated effect sizes of differential methylation within and between studies. Effect sizes from the Braak stage EWAS in the STG from this study were plotted against those in the IFG from the same study (**a**); effect sizes from the Braak stage EWAS in the STG (**b**) and IFG (**c**) from this study were plotted against those in the PFC, TG, EC, and cross-cortex meta-analysis from Smith et al. For panel **b** and **c**, only the correlation and *p* value for the same brain is displayed

Of the 14 significant DMPs associated with pathology in the IFG, 5 were nominally associated with pathology (*p* < 0.05) in the STG. The effect sizes in the STG for the 19 DMPs associated with pathology in either the STG or IFG were correlated with the effect sizes for the same probes in the IFG (*r* = 0.50, *p* = 0.03) (Fig. 2a). A full list of DMPs associated with pathology with p value less than 6.79 × 10^–8^ in either the STG or IFG is available in the Additional file 2: Table S1.

Using the reported significant DMP findings from a recent EWAS meta-analysis [21], our results replicated a subset of reported epigenome-wide significant DMPs. There were 377 unique genome-wide significant CpGs (236, 95, 10, and 220 CpGs associated with Braak stage in the prefrontal cortex (PFC), temporal gyrus (TG), EC, and cross-cortex, respectively) reported, among which 344 CpGs passed QC and were present in this dataset. 236 (68.6%) of these 344 CpGs were nominally significant (*p* < 0.05) in our study, while 22 (6.4%) remained significant after accounting for the multiple tests of the replication effort (*p* < 0.05/344 ~ 0.0001) (Table 2). The replication rate in the IFG seemed to be higher than that in the STG. Eighteen (8.33%) out of 216 significant DMPs in the PFC (216 out of 236 DMPs from the meta-analysis passed QC in this study) were replicated in the IFG analysis, as opposed to 4 (4.49%) out of 89 (89 out of 95 significant DMPs from the TG meta-analysis passed QC) were replicated in the STG analysis. The replicated DMPs included probes annotated to genes in *RMGA*, *GNG7*, *HOXA3*, *GPR56*, *SPG7*, *PCNT*, *RP11-961A15.*1, *MCF2L*, *RHBDF2*, *ANK1*, *PCNT*, *TPRG1*, and *RASGEF1C*. The effect sizes in the STG for the Braak stage association were correlated with those in the meta-analysis except for the EC (Fig. 2b, *r* = 0.77, 0.78, 0.77, all *p* < 2.2 × 10^–16^ for cross-cortex, PFC, TG, respectively; *r* = 0.32, *p* value = 0.40 for EC). The same was true for the effect sizes in the IFG except for the EC when compared to the meta-analysis effect sizes (Fig. 2c, *r* = 0.78, *p* < 2.2 × 10^–16^ for cross-cortex; *r* = 0.77, *p* < 2.2 × 10^–16^ for the PFC; *r* = 0.70, *p* = 3.38 × 10^–14^ for the TG; and *r* = 0.08, *p* = 0.83 for the EC).

### DMR analysis results

A DMR analysis, which allowed us to identify regions of the genome consisting of ≥ 3 probes, revealed a total of 121 and 173 DMRs significantly associated with the pathology in the STG and IFG, respectively (Sidak-corrected *p* value < 0.05, Additional file 2: Table S2A and S2B), among which 11 and 33 were reported to be significant DMRs associated with pathology identified in the corresponding cortex region in the recent EWAS meta-analysis [21]. Lists of replicated DMRs in the same brain region and more broadly in any brain region are available in Additional file 2: Table S3A and S3B. The most striking genomic regions associated with pathology in the IFG are 6 DMRs spanning *HOXA2/HOXA3/HOXA-AS2/HOXA5* consisting of a total of 79 probes (Additional file 1: Figure S3A). This gene cluster is composed of DMRs in *HOXA3* (chr7: 27,153,580–27,155,548 [23 probes], Sidak-corrected *p* = 1.70 × 10^–8^); *HOXA-AS2* (chr7:27,161,749–27,163,095 [11 probes], Sidak-corrected *p* = 3.65 × 10^–9^), *HOXA5* (chr7:27,183,274–27,184,375 [25 probes], Sidak-corrected *p* = 7.62 × 10^–6^); *HOXA2* (chr7:27,143,046–27,143,806 [11 probes], Sidak-corrected *p* = 2.65 × 10^–7^; chr7:27,145,972–27,146,445 [5 probes], Sidak-corrected *p* = 8.86 × 10^–5^; and chr7:27,150,031–27,150,403 [4 probes], Sidak-corrected *p* = 9.65 × 10^–4^). The same gene cluster was identified in the STG with 3 DMRs spanning the *HOXA3/HOXA-AS2/HOXA5* gene cluster [32 probes]. Majority of the probes in the *HOXA* gene cluster were hypermethylated.

In addition, 4 DMRs associated with pathology in the IFG were detected in regions annotated to *MCF2L* consisting of a total of 17 probes (Additional file 1: Figure S3B); the DMR annotated to *MCF2L* was also detected in the STG*.* Furthermore, multiple DMRs annotated to *TFAP2E* (STG), *ZNF608* (STG), *STRA6* (IFG)*, LHX6* (IFG)*, SHH* (IFG)*, and LINC00870* (IFG) were also detected. The *HOXA* gene cluster, *MCF2L, TFAP2E, SHH,* and *LHX6*, was among the replicated DMRs reported previously in the recent EWAS meta-analysis [21]. Additional replicated DMRs supported by a single DMR include regions annotated to *RGMA, CD82, CPEB4, RHBDF2* in both STG and IFG, *C3, CUX2, CLDN5, CXXC5, DDAH2, DIP2A, PARS2*, *S1PR4, SLC16A3, HLA-DPA1, SMG9, ATP2A3, ZNF385A, DUSP27, CAMTA1,* and the *HOXB* gene cluster (Additional file 1: Figure S3C) in the STG*,* and *NAT8L, DDR1, SLC15A4, RHOB* in the IFG (see Additional file 2: Tables S4A and S4B for a full list of replicated DMRs). In total, 26 (16.3%, calculated at the gene level) of the reported 262 significant DMRs (annotated to 160 unique genes) from the PFC meta-analysis were replicated in the IFG analysis, while 15.8% of our 173 significant DMRs (annotated to 164 unique genes) were reciprocally replicated by the PFC meta-analysis. In addition, 11 (18.3%) of the reported 104 significant DMRs (annotated to 60 unique genes) from the TG meta-analysis were replicated in the STG analysis, while 9.2% of our 121 significant DMRs (annotated to 119 unique genes) were reciprocally replicated by the TG meta-analysis. The replication rate at the DMR level seems to be higher than that at the DMP level.

Among the top DMRs associated with pathology in the IFG, the DMR annotated to *DDAH2* stood out as one of the most significant DMRs (chr6:31,695,970–31,696,867 [26 probes], Sidak-corrected *p* = 1.82 × 10^–13^). All of the 26 probes including a genome-wide significant DMP cg25845158 (*p* = 2.55 × 10^–8^ in the IFG) located in the CpG island were hypermethylated (Additional file 1: Figure S3D). A similar DMR (chr6:31,695,973–31,696,729 [21 probes], Sidak-corrected *p* = 1.37 × 10^–5^) associated with pathology was also identified in the STG from this study and in the PFC in the recent EWAS meta-analysis (chr6:31,695,027–31,695,064 [3 probes], Sidak-corrected *p* = 8.13 × 10^–4^). *DDAH2* encodes dimethylarginine dimethylaminohydrolase 2, an enzyme that functions in nitric oxide generation by regulating the cellular concentrations of methylarginines, which in turn inhibits nitric oxide synthase (NOS) activity.

### AD-associated DMRs are enriched/depleted in specific genomic features

**Fig. 3:**
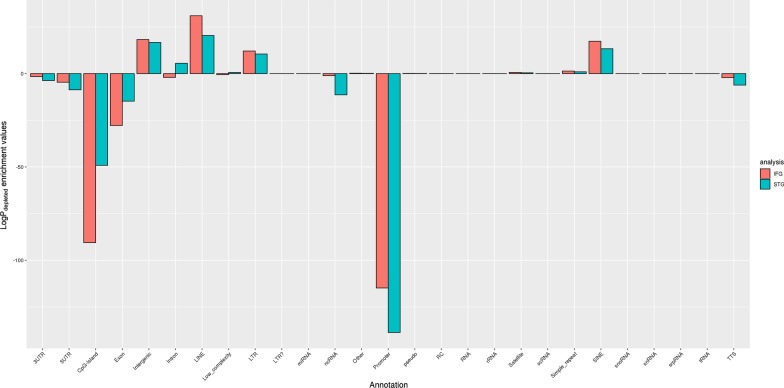
Enrichment of genomic features among the differentially methylated regions (DMRs). The identified DMRs were annotated by HOMER [62]. HOMER first determined the distance of a DMR to the nearest transcription start site (TSS) and assigned the DMR to that gene; it then determined the genomic annotation of the region occupied by the center of the DMR and performed enrichment analysis of genomic features including TTS (transcription termination site), 5′-/3′-untranslated region (UTR), long interspersed nuclear element (LINE), short interspersed nuclear element (SINE), long terminal repeat (LTR), ncRNAs, small nucleolar RNA (snoRNA), small nuclear RNA (snRNA), signal recognition particle RNA (srpRNA), small conditional RNA (scRNA), non-coding RNA (ncRNA), microRNA (miRNA), ribosomal RNA (rRNA), transfer RNA (tRNA), etc.

Among the DMRs that were associated with pathology, genomic features such as promoter and CpG island (CGI) are highly enriched in all analyses. Exon, 5′ UTR, and transcription termination site (TTS) are also enriched. In contrast, intergenic region and repeats (SINE and LINE) are depleted (Fig. 3 and Additional file 2: Table S4).

### Gene set enrichment analysis (GSEA) and over-representation analysis (ORA) results

Gene set enrichment analysis of the DMPs and DMRs revealed that gene sets involved in neuroinflammation, neurogenesis, and cognition were enriched (false discovery rate (FDR) < 0.05. See Additional file 2: Tables S5 and S6 for a full list of enriched gene sets from the DMP and DMR analyses).

Gene sets related to neuroinflammation were enriched in this study. Over representation analysis revealed that genes involved in microglia differentiation (driven by *TSPAN2* and negative regulator of reactive oxygen species (*NRROS*)) were enriched among the DMRs associated with pathology in the STG (Additional file 2: Table S5A). Noticeably, the DMR in *NRROS* was also replicated in the recent EWAS meta-analysis [21]. Phagocytosis (*p* = 0.001, adjusted *p* value = 0.05) was also enriched in DMPs associated with pathology in the IFG (Additional file 2: Table S5B).

For DMPs associated with pathology in the STG, positive regulation of neurogenesis (*p* = 0.001, adjusted *p* value = 0.09) was also enriched (Additional file 2: Table S5A). Likewise, genes involved with neurogenesis were also enriched among the DMRs in the STG (Additional file 1: Table S6). Neurogenesis is critical for learning. Moreover, learning or memory (*p* = 0.001, adjusted *p* value = 0.05) was enriched among DMPs associated with pathology in the IFG (Additional file 1: Table S5B). Over-representation analysis for DMRs associated with pathology in the IFG suggested that genes involved in cognition were enriched (Additional file 2: Table S6), and this enrichment was driven by DMRs in zinc finger protein 385A (*ZNF385A*), CREB-regulated transcription coactivator 1 (*CRTC1*), SH3 and multiple ankyrin repeat domains 2 (*SHANK2*), cut-like homeobox 2 (*CUX2*), cytoplasmic FMR1 interacting protein 1 (*CYFIP1*), claudin 5 (*CLDN5*), stimulated by retinoic acid 6 (*STRA6*), and janus kinase and microtubule interacting protein 1 (*JAKMIP1*). Among these DMRs, *ZNF385A, CUX2,* and *CLDN5* were replicated in the recent EWAS meta-analysis [21].

### Correlation between selected CpG probes and between CpG probes and transcript level

Correlation among top *ANK1* CpG cg13609385 (nominally associated with diagnosis in the STG, *p*_*DMP*_ = 0.001), top *HOXA* gene cluster probe cg14058329, top *HOXB* gene cluster probe cg04904318 (*p*_*DMP*_ = 8.79 × 10^–6^), and top *DDAH2* probe cg25845158, and between these probes and expression level of all transcripts, were tested. Top *HOXB* gene cluster probe cg04904318 was correlated with multiple CpG probes. For example, cg13609385 was positively correlated (*r* = 0.74, *p* = 1.33 × 10^–14^) with cg04904318 in the STG, but negatively correlated with cg04904318 (*r* = −*p* valu0.72, *p* = 2.06 × 10^–11^, Additional file 1: Table S7) in the IFG, suggesting a brain region specificity in their interaction. This correlation is significant even after multiple testing correction (*p* < 0.05/(4*2*(5772+3)) ~ 1.08 × 10^–6^ to correct for testing correlation with 5,772 transcripts and 3 other CpG probes for a total of 4 probes tested in 2 brain regions). In addition, there was a positive correlation between the methylation level of *DDAH2* probe cg25845158 and that of cg04904318 in both the STG and IFG (*r* = 0.549, *p* = 2.77 × 10^–7^ in the STG; *r* = 0.588, *p* = 3.33 × 10^–7^ in the IFG, Additional file 1: Figures S4A and 4B).

## Discussion

In this study, we profiled methylome from the STG and IFG brain regions and attempted to identify evidence of replication of the reported DMPs and DMRs from a recent EWAS meta-analysis [21]. We identified DMRs with replication evidence and replicated 22 significant DMPs reported in the recent EWAS meta-analysis. In addition, we discovered novel DMPs and DMRs associated with pathology surpassing the genome-wide significance threshold (*p* < 6.79 × 10^–8^ for DMPs, and Sidak-corrected *p* value < 0.05 for DMRs) for future follow-up studies.

It is of interest to note that despite the modest sample size, the replication rate for DMRs was substantial. The DMRs identified in this study were supported by more probes than that of previous studies [20, 21], owing to the fact that the EPIC BeadChip used in this study doubles the CpG probe density compared to the previous studies that utilized the HumanMethylation450 BeadChip. This suggests that the higher CpG probe density could increase the power of DMR detection, given comparable study sample size. Indeed, for this study the replication rate at the DMR level is higher than that at the DMP level. 8.33% of the DMPs and 16.25% of the DMRs reported in the PFC meta-analysis were replicated in the IFG analysis, while 4.49% of the DMPs and 18.33% of the DMRs reported in the TG meta-analysis were replicated in the STG analysis. It appears that this study replicated more DMPs identified from the IFG region than the STG region. This could be due to the differential power of the meta-analysis in that the sample size in the PFC meta-analysis is larger than that in the TG meta-analysis (sample size *n* = 959 for the PFC vs. *n* = 608 for the TG), and hence, more (and perhaps also more reliable) DMPs were discovered (*n* = 236 for the PFC vs. *n *= 95 for the TG).

The activation of *HOX* genes during differentiation was enriched among the DMPs associated with pathology in the STG (Additional file 2: Table S5A). This is not surprising given the single genome-wide significant DMP in the *HOXA* gene cluster and multiple significant DMRs in both *HOXA* and *HOXB* gene clusters were identified in this study. Both *HOXA* and *HOXB* differential methylation findings were reported previously in the AD brain [17–21, 32]. Additionally, *HOXA* differential methylation was reported in the blood from patients with Down syndrome [33] and *HOXB* differential methylation was also reported in the blood from patients with AD [34]. Many Down syndrome patients develop AD resulting from an extra copy of the *APP* gene due to trisomy on chromosome 21. In *Drosophila*, it has been shown that the HOX transcription factor is one of the upstream regulators coordinating ankyrin-dependent microtubule organization and synapse stability [35] and it is therefore potentially neuroprotective. *ANK1* was also reported to be differentially methylated in prior studies [16–18, 32] and replicated in this study. We therefore tested the correlation of top *ANK1* CpG cg13609385 and representative probes in the *HOX* gene clusters. Surprisingly, cg13609385 was positively correlated with the lead *HOXB* probe cg04904318 in the STG, but negatively correlated with cg04904318 (Additional file 2: Table S7) in the IFG, suggesting a brain region specificity in their interaction.

Given the role of *DDAH2* in oxidative stress response, hypermethylation of *DDAH2* could theoretically result in lower level of *DDAH2* gene expression and increased NOS activity, excessive reactive oxygen species (ROS) production, and higher level of neuroinflammation, as shown previously that *DDAH2* expression level was inversely correlated with proinflammatory cytokines IL-6 and TNF-alpha [36]. However, with limited overlapping samples (*n* = 76 for the STG samples) between mRNA-Seq and EPIC array samples, we did not have evidence to support the negative correlation between the genome-wide significant DMP cg25845158 from *DDAH2* and *DDAH2* mRNA level, suggesting the transcriptional regulation of *DDAH2* is more complicated than simple regulation by the methylation at the CpG island. In contrast, there was a nominal positive correlation between cg25845158 and *DDAH2* mRNA level in the IFG (*r* = 0.316, *p* = 0.01, Additional file 1: Figure S4C) and no correlation in the STG (Additional file 1: Fig. 4D). Interestingly, there was a positive correlation between the methylation level of *DDAH2* probe cg25845158 and the methylation level of the lead probe in the *HOXB* gene cluster cg04904318 in both the STG and IFG. The significance of this correlation is unknown. Furthermore, *DDAH2* probe cg25845158 was negatively correlated with the transcript level of *SNAP25,* (*r* = − 0.62, *p* = 4.34 × 10^–8^), *MAPK8IP2* (*r* = − 0.59, *p* = 2.63 × 10^–7^), *PDE2A* (*r* = − 0.59, *p* = 2.86 × 10^–7^), and *BZRAP1* (*r* = − 0.57, *p* = 1.01 × 10^–6^) (Additional file 1: Table S7). This is of interest as it is thought that Aβ peptides trigger synaptic dysfunction by interfering with the synaptic vesicular fusion facilitated by the SNARE protein complexes including SNAP25 [37]. *MAPK8IP2* is also known as *JIP2*, c-Jun NH(2)-terminal kinase (JNK)-interacting protein 2, which is known to interact with Aβ to play an important role in the metabolism and/or the function of Aβ including the regulation of Aβ phosphorylation by JNK [38]. Inhibition of PDE2 has been shown to rescue Aβ induced memory impairment via regulation of PKA/PKG-dependent neuroinflammatory and apoptotic pathways [39]. Finally, genetic variants from *BZRAP1-AS1* were previously implicated to be associated with AD [40].

While this study identified interesting genes and pathways, there are limitations that worth commenting. Despite the modest sample size for the two brain regions included in this study, the sample size is still far smaller compared to the pooled sample size in the recent meta-analysis, and hence limits the power in identifying more genome-wide significant DMPs and DMRs. This is a cross-sectional study using samples from the end stage of a disease and therefore it is difficult to infer whether the methylation change is causal or is a result of the disease process. The current study annotates CpG probes to the nearby genes based on the genomic location. It is possible that a regulatory element may interact with another sequence element in the distance via chromatin loop, and therefore, the functional consequence could affect another distal gene. This is a study using DNA extracted from bulk tissue despite the correction for neuronal proportion in the DMP analysis. Studies on cell-type specific methylation pattern [19, 32] revealed cell-type specific effect, which could be obscured in studies using bulk tissues. Finally, this study did not distinguish between 5-mC and 5-hmC and could have missed specific differences between the two. Further studies are needed to replicate the novel DMPs or DMRs identified in this study.

## Conclusions

We conducted a modest size EWAS to identify DMPs and DMRs associated with pathology. Five and 14 study-wide significant DMPs were identified to be associated with pathology in the STG and IFG, respectively. Our study replicated 22 DMPs supporting the findings of a recent EWAS meta-analysis. Additionally, there was substantial overlap between the DMRs identified in this study and those identified in the recent meta-analysis. The identified DMPs and DMRs converged on biological pathways and processes that were previously implicated in AD.

## Methods

### Cohort

Postmortem brain case samples from patients with AD and control samples from patients who were cognitively normal from the STG (*n*_case_ = 91, *n*_control_ = 61) and IFG (*n*_case_ = 89, *n*_control_ = 57) were acquired from Banner Sun Health Research Institute [41, 42]. These brain samples came from subjects who were volunteers in the Arizona Study of Aging and Neurodegenerative Disorders (AZSAND) and the Brain and Body Donation Program, a longitudinal clinicopathological study of healthy aging, cognition, and movement in the elderly since 1996 in Sun City, Arizona.

### Postmortem brain tissue epigenetic profiling

Genomic DNA and total RNA, including miRNA, were simultaneous purified from the brain tissue samples using AllPrep DNA/RNA/miRNA Universal Kit (QIAGEN Inc., Germantown, MD, USA) following the standard protocol. 10 μl of genomic DNA with minimal concentration of 40 ng/μl was bisulfite converted using the Zymo EZ DNA Methylation™ kit (Zymo, Irvine, CA, USA) using the manual protocol, while genome-wide methylation was measured using Infinium® MethylationEPIC BeadChip (Illumina, San Diego, CA, USA) using the automated protocol as detailed in the Infinium® HD Assay Methylation Protocol. Methylome profiling data were generated over two batches for each brain region, respectively. All data generation were conducted by laboratory personnel blinded as to the clinical phenotype.

### Postmortem brain tissue mRNA-Seq

The mRNA-Seq study was reported previously [43]. RNA samples (*n*_case_ = 24, *n*_control_ = 38) from the same cohort above with RNA integrity number (RIN) greater than 6 were proceeded to the library construction step for mRNA-Seq data generation. Libraries were constructed using TruSeq® Stranded mRNA Library Prep (Illumina Inc., San Diego, CA, USA) according to manufacturer’s protocol using 200 ng of input RNA. Briefly, poly-A-containing mRNA was captured using poly-T oligonucleotide-attached magnetic beads. Following purification, the mRNA was fragmented using divalent cations under elevated temperature. The cleaved RNA fragments were copied into first strand cDNA using reverse transcriptase and random primers. Strand specificity was achieved by replacing dTTP with dUTP in the Second Strand Marking Mix (SMM), followed by second strand cDNA synthesis using DNA polymerase I and RNase H. These cDNA fragments were then followed by A-tailing and adapter ligation reactions. The products were purified and enriched with PCR to create the final cDNA library. All libraries were quantified by Caliper and real-time qPCR and amplified on cBot to generate the clusters on the flowcell, and sequenced using HiSeq4000 (Illumina Inc., San Diego, CA, USA) using paired end (100 bp × 2) sequencing to a sequencing depth of 40M reads (or 8G data). Sequencing data were generated over two batches for each brain region. All data generation were conducted by laboratory personnel blinded as to the clinical phenotype. This dataset was used to perform correlation analysis of selected CpG probes and mRNA transcript to shed light on the potential consequence of DNA methylation.

### Data Pre-processing

Epigenetic data were analyzed separately for each brain region/wave. Quality control of the epigenetic data was performed using ChAMP R package [44]. Probes that did not perform well (with detection *p* value ≥ 0.01 in one or more samples (*n*_STG_ = 10,136 and *n*_IFG_ = 10,920 for the STG and IFG samples, respectively), or with bead count < 3 in at least 5% of samples (*n*_STG_ = 4220 and *n*_IFG_ = 7179), probes with known SNP sites or with cross-reactivity [45] (*n*_STG_ = 95,414 and *n*_IFG_ = 94,915), non-CG probes (n_STG_ = 2910 and n_IFG_ = 2900), probes align to multiple locations on the genome [46] (*n* = 15), as well as probes located on the sex chromosomes (*n*_STG_ = 16,400 and *n*_IFG_ = 16,287) were filtered out. At the sample level, gender based on the methylation data was estimated using getSex function in the minfi (v1.28.4) R package and compared to that from the clinical phenotype. No discrepant gender was detected for the study samples. Since a subject may have samples from two brain regions assayed in this study, sample identity check was performed using R package ewastools (v1.6) [47]. All expected pairs of identity were confirmed, and all detected pairs of identity were expected.

The methylation levels were then normalized using Dasen method in R package wateRmelon [48]. The neuronal vs. non-neuronal cell composition was estimated using the estimateCellCounts function in minfi [49] which used a reference brain dataset of fluorescence activated cell sorting (FACS) sorted neuronal and non-neuronal nuclear fractions [50]. Surrogate variables are covariates constructed directly from high-dimensional data that could be used in subsequent analyses to adjust for unknown, unmodeled, or latent sources of noise [51, 52]. We used sva (v3.30.1) [53, 54] to detect and estimate surrogate variables for unknown sources of variation to remove artifacts in the high-throughput experiments. Removing batch effects using surrogate variables in differential analysis have been shown to reduce dependence, stabilize error rate estimates, and improve reproducibility [55]. Samples with discrepant phenotype between sample label and the phenotype data linked to case ID on the sample label were excluded from downstream analysis. To have a balanced age-matched study design, only samples from subjects aged between 60 and 89 inclusive were included in the analysis resulting in sample sizes of 127 samples (67 AD and 60 cognitively normal control) and 117 samples (60 AD and 57 cognitively normal control) for the STG and IFG, respectively, used in downstream analysis.

mRNA-Seq data were processed per sample using cutadapt (v1.13) [56], and STAR (v2.5.3a) [57]. Transcript quantification was performed using RSEM (v1.3.0) [58] against all 26,000 genes in NCBI RefSeq database (version date; 2015-07-17).

### Identification of DMPs

We used M value in the statistical analysis to identify DMPs using limma [59] as M value was shown to provide better performance in detection rate and true positive rate for both highly methylated and unmethylated CpG sites and was more statistically valid than beta-value, despite beta-value was more biologically intuitive [60]. Epigenetic association model corrected the top five surrogate variables, sex, age, neuronal proportion, and Braak stage (as a continuous variable) was tested in a linear regression model to identify differentially methylated probes associated with Braak stage. Epigenome-wide association studies were prone to significant inflation and bias of test statistics, and a Bayesian method to control bias and inflation in EWAS based on estimation of the empirical null distribution was proposed and implemented in R package BACON [30]. We applied this Bayesian method as implemented in BACON v1.10.1 to control for inflation and lambda (l) inflation factors before and after correction was reported. A stringent threshold using Bonferroni correction was used to declare study-wide significance.

The discovered DMPs in each brain region were examined for consistency evidence in several ways. Firstly, we checked for consistency of effect size and directionality between the two brain regions in this study; secondly, we compared the effect sizes from this study to those reported in the recent meta-analysis [21]. Lastly, we attempted to replicate the published genome-wide significant DMPs and DMRs from the recent meta-analysis given that our data were generated using Illumina EPIC BeadChip, and the published studies were using the Illumina HumanMethylation450 BeadChip.

### Identification and annotation of DMRs and genomic feature enrichment

DMRs were identified using comb-p [61] with a distance of 500 bp and a seeded *p* value of 1.0 × 10^–4^. The DMR analyses were carried out for all probes (irrespective of directionality of differential methylation), and DMRs with at least three probes and Sidak-corrected *p* less than 0.05 were considered significant and reported. The identified DMRs were annotated by HOMER [62]. HOMER first determined the distance of a DMR to the nearest transcription start site (TSS) and assigned the DMR to that gene, it then determined the genomic annotation of the region occupied by the center of the DMR and performed enrichment analysis of genomic features.

### Gene set enrichment analysis

ORA [63] for genes near significant CpGs from Illumina's Infinium Human MethylationEPIC array was performed using missMethyl R package v1.16.0 [64], taking into account the differing number of probes per gene present on the array. Additionally, a GSEA [65] analysis was performed using R package methylGSA [66] adjusting for multiple *p* values of each gene by Robust Rank Aggregation (RRA), and then apply pre-ranked version of GSEA (GSEAPreranked) in gene set testing. Lastly, methylglm function within R package methylGSA was used for length bias correction using logistic regression [67].Gene ontology databases used included KEGG database [68] and c2.cp (a superset of c2.cp.biocarta, c2.cp.kegg, and c2.cp.reactome [69] and a few other data sources) (v7.0) subsets of Molecular signatures database (MSigDB) [70].

Over-representation analysis of genes implicated by DMR was performed using https://www.gsea-msigdb.org/gsea/msigdb/compute_overlaps.jsp which has a broader background gene set assumption and test over-representation at higher levels of the ontology hierarchy. Gene ontology databases used included c2.cp and c5 subsets of MSigDB [70].

### Methylation-mRNA correlation analysis

In order to identify potential consequence of DNA methylation, paired methylation level-mRNA correlation analysis was performed using Partek Genomic Suite (Partek Inc, St Louis, MO, USA), which only examined the correlation between selected top CpG probes (*n* = 4) and the transcript level. We used fsva function in sva R package to perform frozen surrogate variable analysis [71] to remove nuisance batch effects from both methylation array and mRNA-Seq datasets and used the adjusted version of datasets for correlation analysis. Multiple testing correction was applied (*p* < 0.05/(4*2*(5772 + 3)) ~ 1.08 × 10^–6^ to correlate for testing correlation with 5772 transcripts (only test the moderate to abundant transcripts) and 3 other CpG probes for 4 probes in 2 brain regions) as some of top CpG probes are in the homeobox transcription factors, and the functional consequence could be reflected in the targets of the transcriptional factors.

## Supplementary information

**Additional file 1.** Supplemental Figures S1–S4.

**Additional file 2.** Supplemental Tables S1–S7.

## Data Availability

The EPIC array dataset generated and analyzed during the current study is available from GEO under the accession number GSE156984. The code snippets associated with this manuscript are available at https://github.com/qserenali/EWAS.

## Abbreviations

AD: Alzheimer's disease
EWAS: Epigenome-wide association study
STG: Superior temporal gyrus
IFG: Inferior frontal gyrus
EC: Entorhinal cortex
FDR: False discovery rate
DMP: Differentially methylated position
DMR: Differentially methylated region
GWAS: Genome-wide association studies
NGS: Next generation sequencing
5-mC: 5-Methylcytosine
5-hmC: 5-Hydroxymethylcytosine
NFT: Neurofibrillary tangle
DEG: Differentially expressed gene
GEO: Gene expression omnibus
GSEA: Gene set enrichment analysis
ORA: Over-representation analysis
RIN: RNA integrity number
FACS: Fluorescence activated cell sorting
RRA: Robust rank aggregation
MSigDB: Molecular signatures database
