# Supplementary material for: Epigenome-wide association study of Alzheimer’s disease replicates 22 differentially methylated positions and 30 differentially methylated regions
Source: Clin Epigenetics. 2020 Oct 17;12:149. doi: 10.1186/s13148-020-00944-z (PMC7568396; doi:10.1186/s13148-020-00944-z)
Supplement: Supplementary file 1 — Additional file 1. Supplemental Figures S1–S4. [file 13148_2020_944_MOESM1_ESM.docx]

Epigenome-wide association study of Alzheimer’s disease replicates 22 differentially methylated positions and 30 differentially methylated regions

Qingqin S. Li^1^, Yu Sun^1,a^, Tania Wang^2,b,c^

^1^ Neuroscience, Janssen Research & Development, LLC, 1125 Trenton-Harbourton Road, Titusville, NJ 08560; ^2^AccuraScience, LLC

Current affiliations:

^a^Discovery Science, Janssen Research & Development, LLC, Spring House, PA, USA

^b^Center for Medical Genetics & Hunan Key Laboratory of Medical Genetics, School of Life Sciences, Central South University, Changsha, 410083, China

^c^Beijing Institutes of Life Science, Chinese Academy of Sciences, Beijing 100101, China

**Supplemental Information**

**Figure S1** QQ plots for AD pathology in the STG (a) and IFG (b) before and after BACON correction.

**Figure S2** EWAS Manhattan plots for AD pathology in the STG (a) and IFG (b). Horizontal line depicts association p-value fulfilling Bonferroni correction threshold.

**Figure S3** Mini-Manhattan plots for significant DMRs in (a) the *HOXA* gene cluster (b) *MCF2L* (c) the *HOXB* gene cluster (d) *DDAH2*

**Figure S4** Correlation between CpG probes or between CpG probe and the paired mRNA expression level

**Table S1** CpG sites associated with AD pathology in either the STG or IFG with p_DMP_ < 6.79 x 10^-8^

**Table S2** A list of significant DMRs associated with Braak stage with Sidak corrected P-value less than 0.05

**Table S3** A list of DMRs replicated in the Smith et al., 2020 meta-analysis

**Table S4** Genomic features enriched or depleted among the identified DMRs

**Table S5** Gene sets enriched in the DMPs (padj < 0.1) associated with Braak stage in the (a) STG or (b) IFG in this study.

**Table S6** A list of gene sets enriched in DMRs associated with Braak stage

**Table S7** A list of CpG probes and mRNA transcripts correlated or anti-correlated with the query probe

**Figure S1** QQ plots for AD pathology in the STG (a) and IFG (b) before and after BACON correction.

1. STG


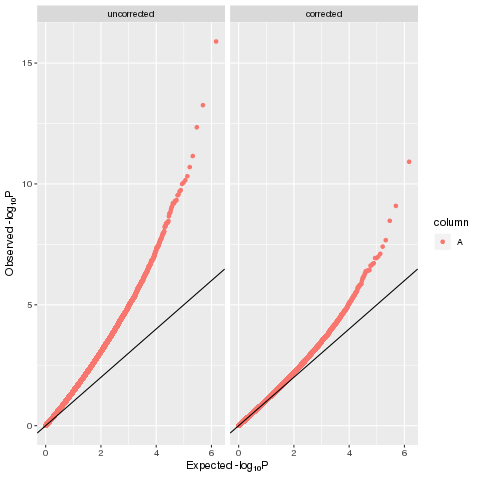


λ = 1.542 (uncorrected); λ = 1.039 (corrected)

1. IFG


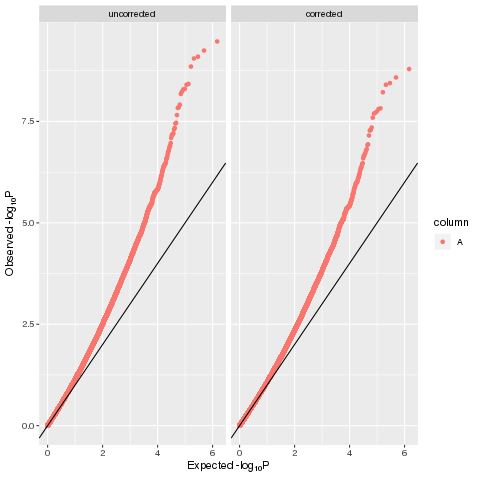


λ = 1.11 (uncorrected); λ = 1.034 (corrected)

**Figure S2** EWAS Manhattan plots for AD pathology in the STG (a) and IFG (b). Horizontal line depicts association p-value fulfilling Bonferroni correction threshold.

1. STG


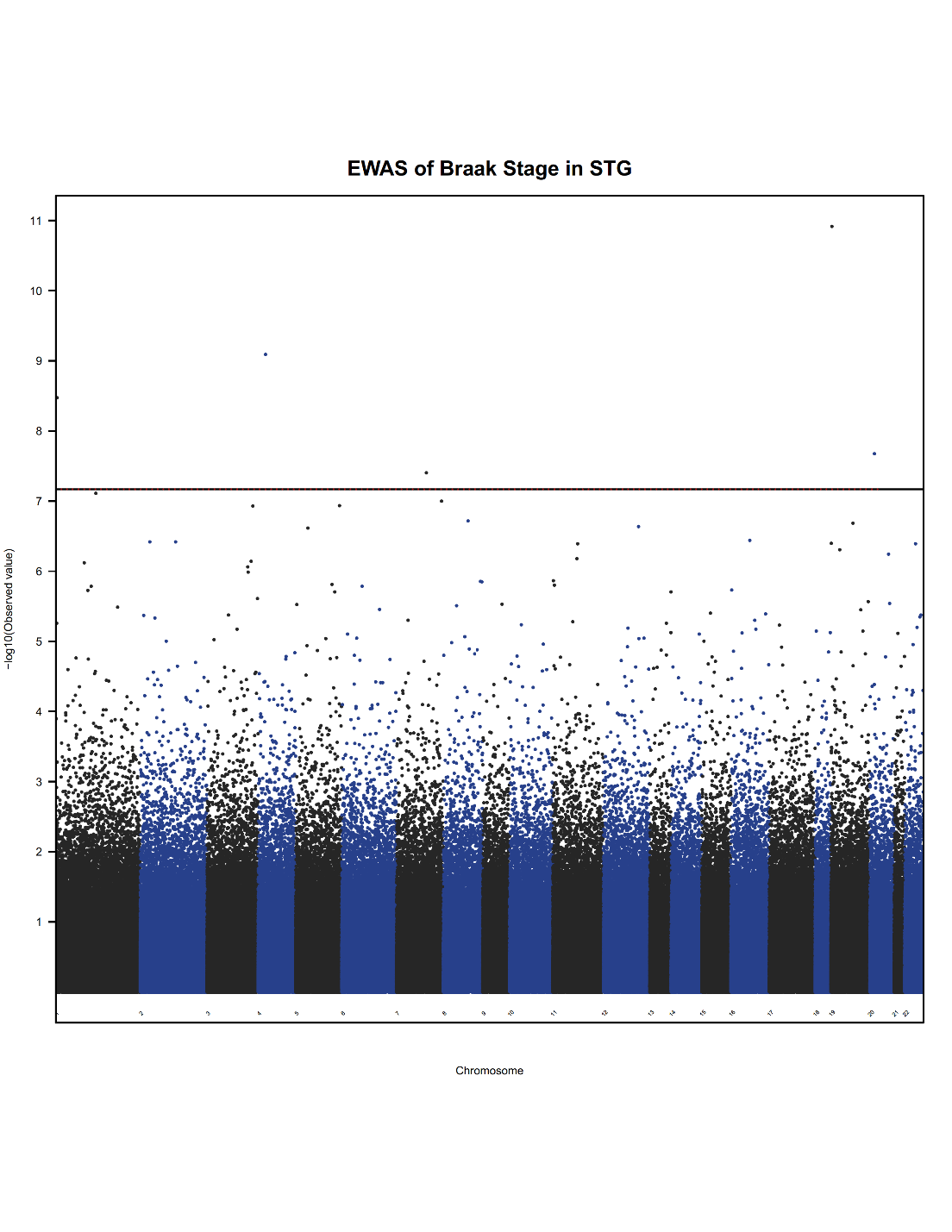


1. IFG

**
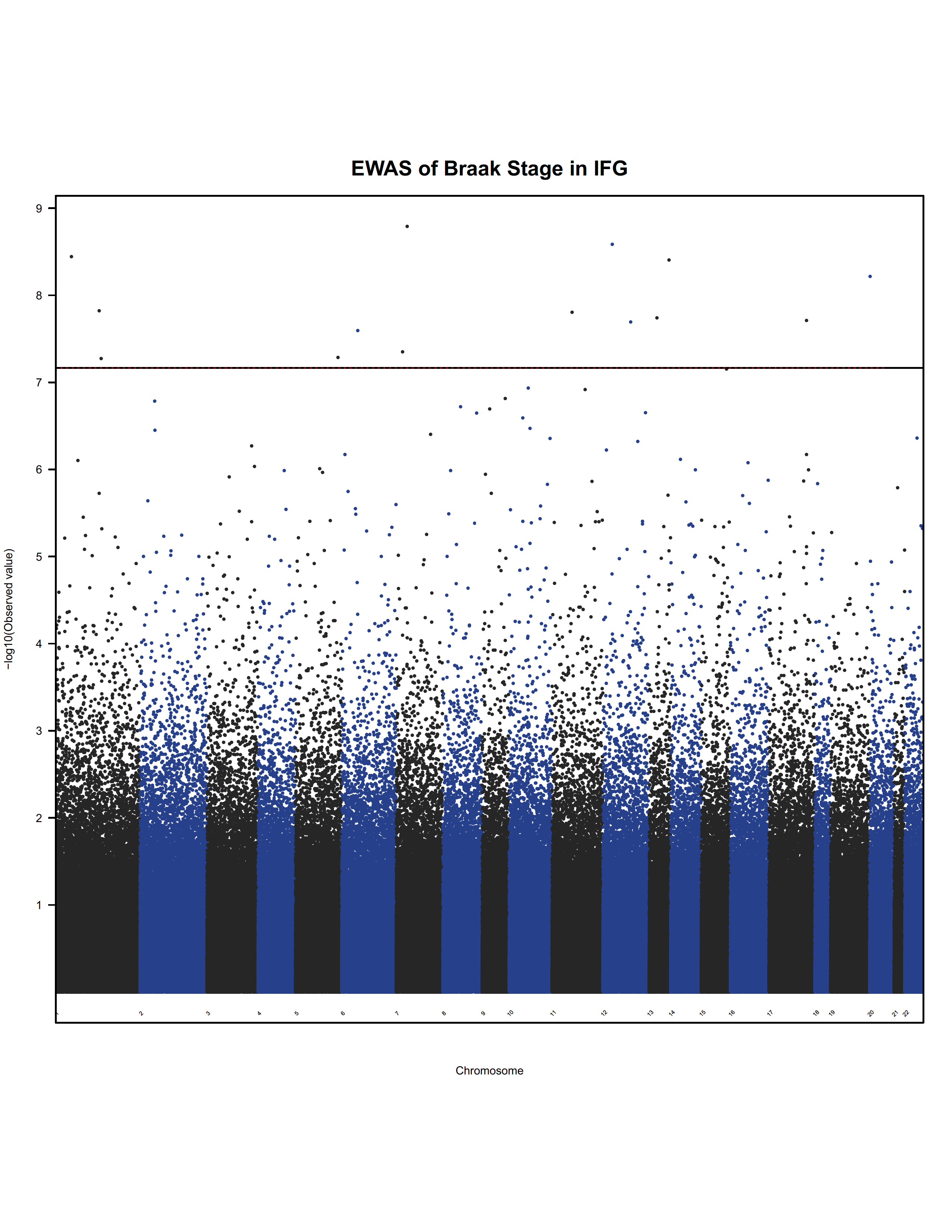
**

**Figure S3** Mini-Manhattan plots for significant DMRs in (a) *HOXA* gene cluster (b) *MCF2L* (c) *HOXB* gene cluster (d) *DDAH2* where color of 1 represents hyper-methylation and -1 represents hypo-methylation. Horizontal blue dash line denotes genome wide significant threshold for DMP analysis.

1. *HOXA* gene cluster


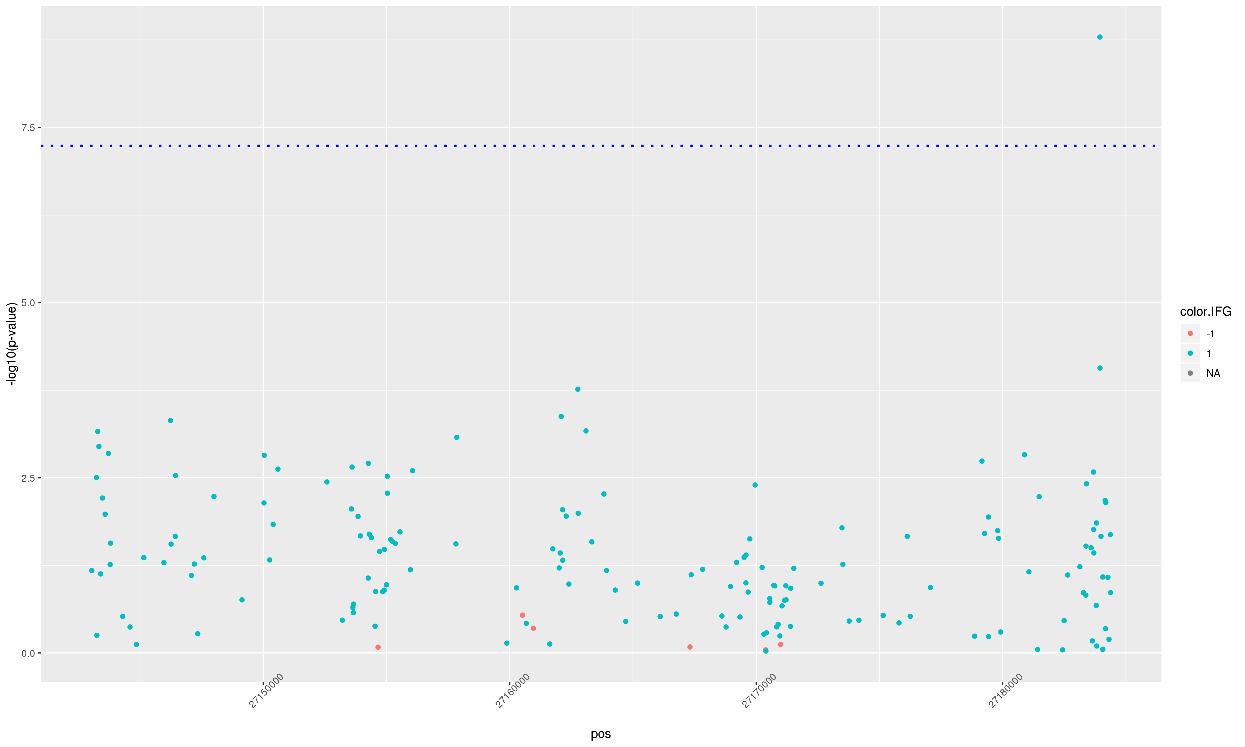


1. *MCF2L*


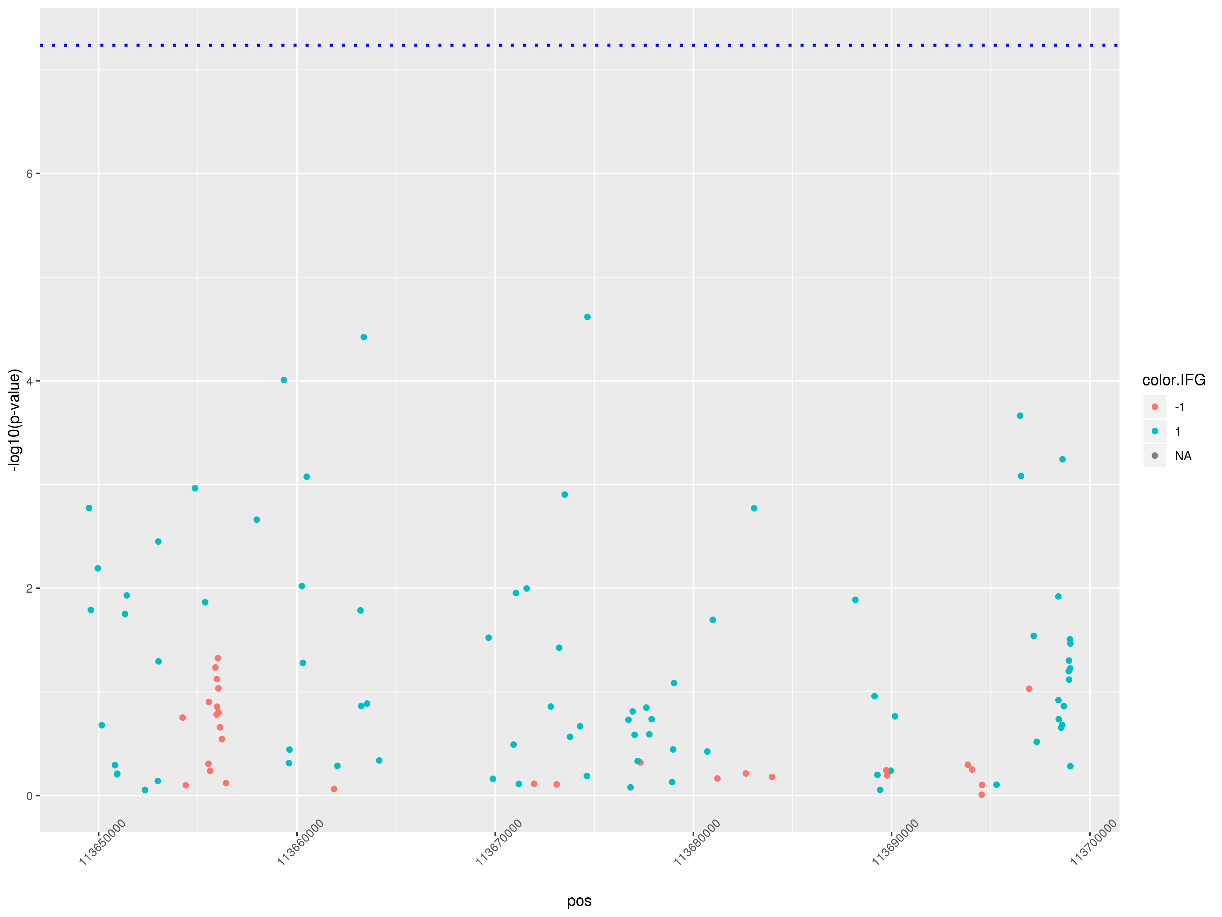


1. *HOXB* gene cluster

**
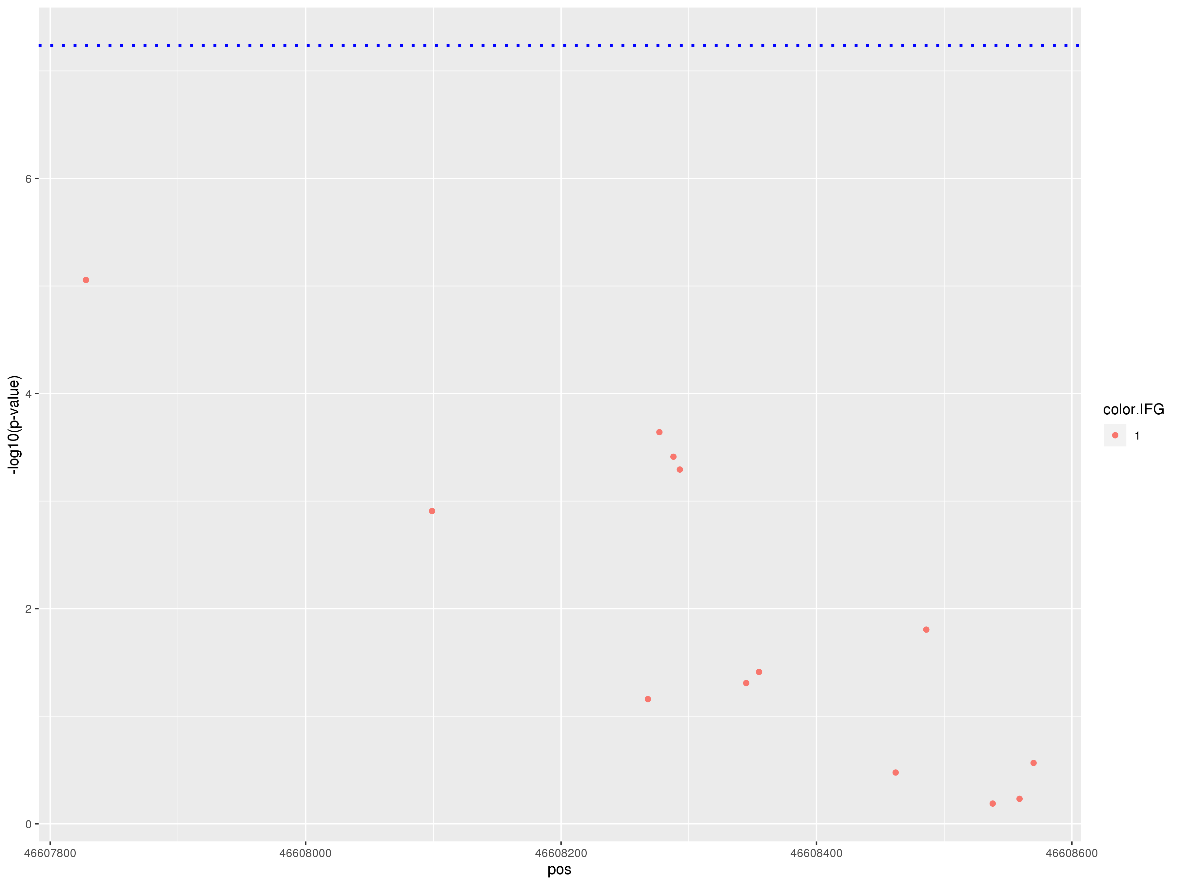
**

1. *DDAH2*

**
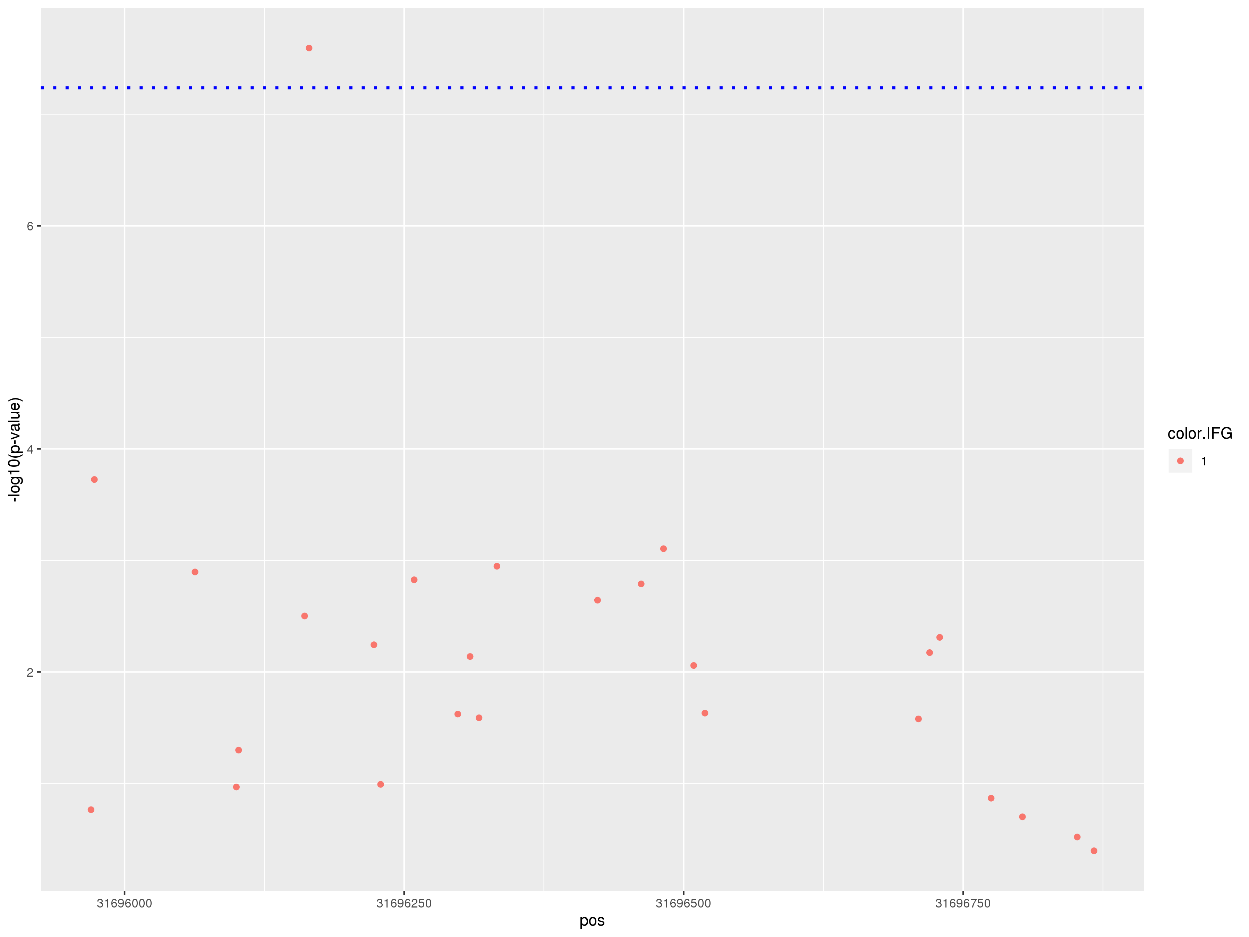
**

**Figure S4** Correlation between CpG probes or between CpG probe and the paired mRNA expression level

1. *HOXB* gene cluster probe cg04904318 vs *DDAH2* probe cg25845158 in the STG


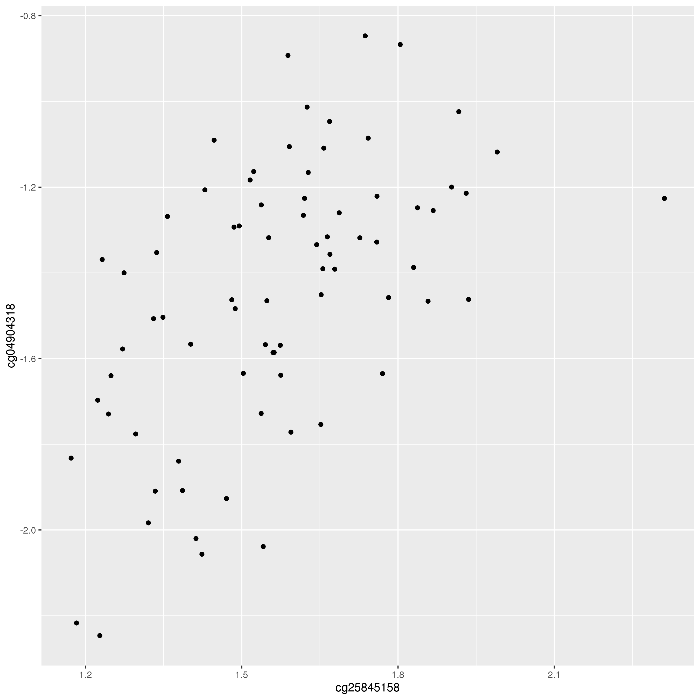


r = 0.549, *p* = 2.77 x 10^-7^

1. *HOXB* gene cluster probe cg04904318 vs *DDAH2* probe cg25845158 in the IFG


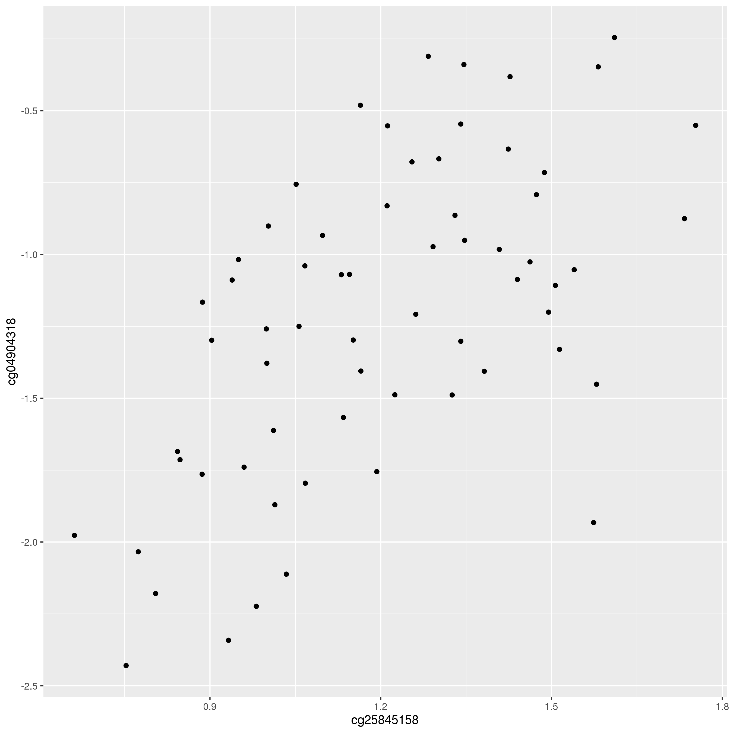


r = 0.588, *p* = 3.33 x10^-7^

1. *DDAH2* probe cg25845158 in CpG island vs. *DDAH2* mRNA level in the IFG


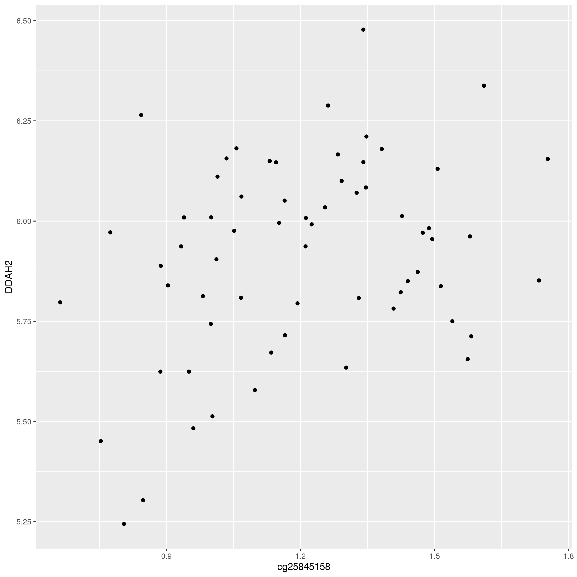


r = 0.316, *p* = 0.01

1. *DDAH2* probe cg25845158 in CpG island vs. *DDAH2* mRNA level in the STG


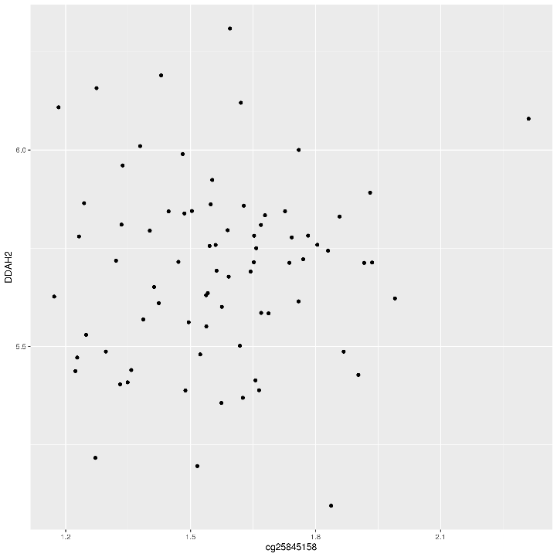


r = 0.061, *p* = 0.60
